# The mechanisms underlying olanzapine-induced insulin resistance via the brown adipose tissue and the therapy in rats

**DOI:** 10.1080/21623945.2022.2026590

**Published:** 2022-01-22

**Authors:** Jing Wang, Qian Wu, Yuan Zhou, Liangyu Yu, Lixiu Yu, Yahui Deng, Chuyue Tu, Weiyong Li

**Affiliations:** a Department of Pharmacy, Union Hospital, Tongji Medical College, Huazhong University of Science and Technology, Wuhan, China; b Department of Pharmacy, Wuhan Xirui Pharmaceutical Technology Co Ltd, Wuhan, China

**Keywords:** Gegen Qinlian Decoction, olanzapine, insulin resistance, brown adipose tissue

## Abstract

A rapid increase has been observed in insulin resistance (IR) incidence induced by a long-term olanzapine treatment with no better ways to avoid it. Our study aimed to demonstrate the mechanism underlying the olanzapine-induced insulin resistance and find appropriate drug interventions. In this study, firstly, we constructed rat insulin resistance model using a two-month gavage of olanzapine and used the main active ingredient mixture of Gegen Qinlian Decoction for the treatment. The activity of brown adipose tissue (BAT) was measured using the PET/CT scan, whereas Western blot and quantitative real-time PCR were used to detect the expression of GLUT4 and UCP1. The results showed that the long-term administration of olanzapine impaired glucose tolerance and produced insulin resistance in rats, while Gegen Qinlian Decoction could improve this side effect. The results of the PET/CT scan showed that the BAT activity in the insulin-resistant rats was significantly lower than that of the Gegen Qinlian Decoction treated rats. Also, the expression of GLUT4 and UCP1 in the insulin resistance group showed a significant decrease, which could be up-regulated by Gegen Qinliane Decoction treatment. The results of both in vivo and in vitro experiments were consistent. we demonstrated that the olanzapine could induce IR in vitro and in vivo by decreasing the expression of UCP1; thus, suppressing the thermogenesis of BAT and impairing glucose uptake. More importantly, we demonstrated a possible novel strategy to improve the olanzapine-induced IR by Gegen Qinlian Decoction.

## 1. Introduction

Second-generation antipsychotics (SGAs) are commonly used drugs in clinical practice because of their good clinical effects on schizophrenia negative symptoms [1]. However, patients who take SGAs for a long time often have metabolic side effects such as weight gain, insulin resistance, and type 2 diabetes, which seriously affect their quality of life [2]. Different affinities of SGAs on dopamine, serotonin, muscarinic, adrenergic, histamine receptors, and other molecular targets (e.g., AMPK) are responsible of their diverse clinical profiles [3]. There is a correlation between SGA’ s clinical efficacy and likelihood of metabolic alterations, whereas drugs with the highest activity in terms of number of receptor targets are the ones with the major risk of metabolic syndrome (MetS). Olanzapine and clozapine are associated with the greatest risk of MetS; however, these aspects must be considered along with their higher efficacy. The newer, AAPs such as ziprasidone, lurasidone are more tolerable on the metabolic profile, but their overall clinical efficacy is less compared with clozapine and olanzapine [4,5]. Reports also have shown that olanzapine (OLZ) is currently one of the leading SGAs sold in China, with a 30% compound annual sales growth rate [6]. So, it is very important to clarify the mechanism underlying the insulin resistance caused by the long-term usage of olanzapine and to find appropriate drug interventions.

**Figure 1.**
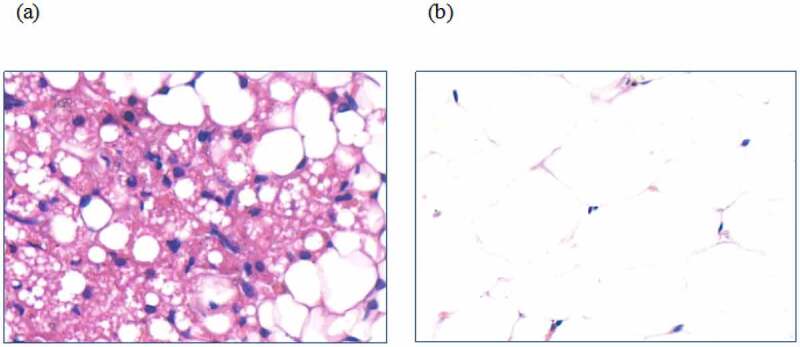
The images of iBAT and scWAT (a) H&E staining of the iBAT; (b) H&E staining of the scWAT.

The adipose tissue in humans is divided into white adipose tissue (WAT) and brown adipose tissue (BAT). WAT is a type of fat responsible for storing energy [7], while BAT can break down WAT and convert it into heat that is released off. Also, it does not store energy and is brown in colour with abundant sympathetic nerve tissues and capillaries [8]. Electron microscope imaging showed that compared to WAT, there were a lot of mitochondria in the BAT. As shown in Figure 1, Active BAT is present in the interscapular area of adults, which plays an important role in the regulation of energy homoeostasis and blood glucose [9–11]. Studies have shown that BAT mainly produces heat through uncoupling protein 1 (UCP1). The intracellular decoupling of UCP1 prevents energy from being converted into ATP and releases it directly as heat [12,13]. UCP1 is one of the in vivo markers of BAT activity, whereas, for the identification of BAT in vitro, currently, ^18^F-FDG-PET/CT imaging technology is majorly used [14]. Also, according to a report, BAT was thought to be involved in OLZ-induced insulin resistance [15].

Gegen Qinlian Decoction (GQD), used as a traditional Chinese herbal medicine, was first discovered in Zhang Zhongjing’s Treatise on Febrile Diseases. It is mainly composed of Pueraria lobata, Scutellaria, Coptis, and Liquorice. Usually, it is used to treat diarrhoea, enteritis, and general fever [16]. Recent studies have found that GQD also has a good therapeutic effect on diabetes, and its blood glucose regulation effect has been confirmed by multiple studies with no reported clinical adverse effects [17]. However, the mechanism of action involved is very complicated, and there is no definite answer for it [18,19]. Zhang et al. conducted on 3T3-L1 cells, serum with GQD could significantly increase the triglyceride (TG) content, adiponectin protein concentration, and adiponectin mRNA expression, suggesting its efficacy in metabolism [20]. Sui et al. found that GQD could improve insulin resistance, possibly by up-regulating SIRT1 and inhibiting Ac-FOXO1 levels in HepG2 cells, which was stimulated by palmitic acid [21]. In this study, we confirmed the effect of GQD in improving insulin resistance and also discovered its possible mechanism of action.

## 2. Materials and methods

### 2.1. Animals and sample collection

Female Sprague–Dawley (SD) rats (200–220 g) (purchased from the Experimental Animal Research Center of Hubei Province, Wuhan, China, animal licence number: SCXK Wuhan 2015–0018) were individually housed in the cages (with conditions of 23–25°C temperature and 40%-60% relative humidity) with a cycle of 12-h light/dark. They had free access to tap water and food. The HUST Animal Ethics Committee specifically approved this study.

After one week of acclimatization, rats were randomly divided into three groups, blank (BL) group, OLZ group, and OLZ+GQD group. The OLZ group was given 1.5 mg/kg of olanzapine (LRAA9505, Sigma, USA) by gavage every day. The BL group was given the same amount of normal saline while the OLZ+GQD group was given olanzapine along with the main active ingredient mixture of Gegen Qinlian Decoction of 310.7 mg/kg/day, using the ratio of puerarin (RYGES20190609, Xi’an Ruiying Biotechnology Co., Ltd, China): berberine (RYHLS20190508, Xi’an Ruiying Biotechnology Co., Ltd, China): baicalin (RYHQG20190609, Xi’an Ruiying Biotechnology Co., Ltd, China): glycyrrhizin (RYGCHT20190523, Xi’an Ruiying Biotechnology Co., Ltd, China) = 6:5:8:1 with warm water [22]. The drugs were administered continuously for eight weeks between 8:00–8:30 am daily, during, which the data for weight were also recorded. The oral glucose tolerance test (OGTT) was performed after the 8th week of administration, where the rats were given a 50% glucose solution with 2.5 g/kg as the limit. Two days after the test, rats were anesthetized, and the interscapular brown adipose tissue and subcutaneous inguinal white adipose tissue were taken out. The tissues were rinsed three times with normal saline solution and quickly frozen in liquid nitrogen. Finally, the samples were stored at – 80°C for subsequent use.

### 2.2. Biochemical estimations

Every week during the test period, plasma was collected from the rats after a fast of 12 h, and then the following measurements were performed: blood glucose level was measured (Cat: F006, Nanjing Jiancheng, China), insulin concentration was measured using a kit (Cat: ELK2370, ELK Biotechnology, China), and the homoeostasis model assessment of insulin resistance (HOMA-IR) was calculated using the formula (HOMA-IR = fasting blood glucose (mmol/L)×fasting insulin (mU/L)/22.5) [23].

### 2.3. PET/CT scan

The rats were scanned using the integrated [^18^F] fluorodeoxyglucose positron emission tomography-computed tomography (^18^F-FDG PET/CT) at 4 and 8 weeks after the administration (lnliView-3000B small-animal PET/SPECT/CT system, Novel Medical Co., Ltd, China). Rats were randomly selected from each group, and fasted for 12 h. Then intraperitoneal injection of 0.4 mg/kg of norepinephrine (190,606, Grandpharma Co., Ltd, China) were given to them. After 1 h, an ^18^F-FDG tracer (Taken from the Medicine Preparation Room of PET Center, Tongji Medical College, Huazhong University of Science and Technology, 500–700 µCi/only) was injected through their tail vein, and during this time they could even drink and walk freely. 18 min after the tracer was injected, the rats were pre-anaesthesia. After 2 min, the rats were transferred to the imaging bed of the animal PET/CT imaging system for fixation. After acquiring CT images at 50 kV and 0.5 mA with a total number of 360 frames, and a slice thickness of 0.18000 mm, a whole-body PET scan was acquired. The PET system parameters were: stop time used was 10 min; the number of slices was 112; slice thickness was 0.80000 mm; the number of iterations used was 40. Finally, to check and analyse the reconstructed CT and PET images, the manufacturer’s software was used.

### 2.4. Pharmacokinetic study

After fasting for 12 h in the evening after the OGTT, the OLZ group and OLZ+GQD group were given drugs the next morning at 8 am. Plasma was collected before administration and at different intervals after administration, including 10 min, 20 min, 30 min, 45 min, 1 h, 2 h, 4 h, 6 h, and 8 h. The samples were further frozen at – 80°C for subsequent testing.

The concentration of olanzapine in plasma was determined using LC/MS/MS (Agilent 1200 liquid chromatography system, Agilent, USA; API4000 QTRAP MSMS system, Applied Biosystems, USA; data acquisition: Analyst 1.6.1, Applied Biosystems, USA) with Butinib (S021409140601, Hengrui Medicine Co., Ltd, China) as the internal standard. The chromatographic conditions were as follows: a Welch Ultimate XB C18 column of 2.0 × 50 mm, 5 µm (Agilent, USA) was used; the top column used was AJ0–4287 of 4 × 3.0 mm (Agilent, USA); the mobile phase was acetonitrile (A): 5 mM ammonium acetate (containing 0.05% ammonia) (B) = 59:41; the flow rate was 0.30 mL/min; autosampler temperature was set at 4°C; column temperature was 35°C; injection volume was 5 µL.

Pre-treatment of the blood sample: 50 µL of internal standard (Ibrutinib, 100 ng/mL) was added to 50 µL of the blood sample. Next, 950 µL of methanol was added to the sample and vortexed for 50s. The sample was then centrifuged at high speed (14000rpm) for 3 min, and the supernatant was collected for LC/MS/MS analysis. The injection volume used was 5 µL.

LC/MS/MS results were analysed using DAS3.0 for pharmacokinetics. The parameters used were as follows: a non-compartmental model with a single dose of 1.5 mg/kg, administered as a non-intravenous injection.

### 2.5. Western blot

The total protein concentration in the samples was determined by the bicinchoninic acid (BCA, Beyotime Biotechnology, China) method. 5× buffer (Tris-HCl, DTT, SDS, BPB, glycerine) solution was added to the samples and heated at 100°C for 10 min. Then, 40 µg of protein from each sample was loaded onto 10% sodium dodecyl sulphate-30% polyacrylamide gels, and electrophoresis was performed. The protein samples were then transferred from the gel onto the nitrocellulose (NC) membranes. The PVDF membrane was incubated with TBST (blocking solution) containing 5% skimmed milk powder and blocked for 2 h at room temperature. The primary antibodies, UCP1 (DF7720, Affinity Biosciences, OH, USA) and Glut4 (AF5386, Affinity Biosciences, OH, USA), were diluted using the blocking solution and then added to the membrane and incubated overnight at 4°C. The excess primary antibody was washed off, and the secondary antibody was added to the membrane with blocking solution and further incubated for 2 h in a shaker at 37°C. Blots were exposed and developed using the enhanced chemiluminescence (ECL) method, while the images were captured and analysed using the image pro plus (IPP 6.0) software.

### 2.6. Cells and reagents

The mouse pre-brown adipocytes were purchased from Procell Life Science & Technology Co. Ltd (Wuhan, China) and 3T3-L1 mouse embryo fibroblasts were purchased from Wuhan HYcell Biotechnology Co., Ltd. (Wuhan, China). Dexamethasone (DEX) was purchased from Selleck (Cat: S1322, Shanghai, China),Liothyronine (T3)was purchased from Wuhan HYcell Biotechnology Co., Ltd (Cat:HY-A0070A,Wuhan, China); 3-isobutyl-1-methylxanthine was obtained from MedChemExpress LLC (Cat: HY-12318, Shanghai, China), and insulin was purchased from Wuhan HYcell Biotechnology Co., Ltd. glucose transporter 4 (GLUT4) (Cat: ab216661, Abcam,1:10), Uncoupling protein 1 (Cat: ab209483, Abcam, 1:10), and GAPDH (Cat: ab37168, Abcam; 1:10,000). All the primary antibodies were raised in rabbits. The secondary antibody used for the Western blot analysis was HRP-goat anti-rabbit (Cat: AS1107, Aspen Biotechnology Co., Ltd., Wuhan, China; 1:10,000). To determine GLUT4 protein expression, the cell membrane GLUT4 assay was conducted using a membrane protein extraction kit.

The 3T3-L1 fibroblasts cells were cultured in the DMEM supplemented with 4 mM L-glutamine, 4.5 g/L glucose, and 10% FBS in a 5% CO_2_ incubator. After two days of fusion, the cells were stimulated to synthesize fat by changing the medium to DMEM containing 1 mM DEX, 25 mM glucose, **0.5 mM IBM-X,** 10 mg/mL insulin, and 10% FBS. The cells were then maintained in 10% FBS/DMEM medium with 10 mg/mL insulin for two days. After 46 h, the medium was replaced with DMEM/high glucose. During this 10–14 day experiment, the medium was changed every two days until 90–95% of the cells showed the phenotype of adipocytes. Differentiation was monitored visually by the appearance of fat droplets inside the cells.

The brown pre-adipocytes were cultured in an incubator at 37°C with 5% CO_2_ and saturated humidity of 100% confluence. Contact inhibition occurred two days after the cells became fully confluent. 20 nM of insulin, 1 nM of, 0.5 mM of IBMX, 0.5 µM DEX were then added. A medium containing 0.5 µM DEX 0.125 mM indomethacin, 10% foetal bovine serum, and 1% penicillin/streptomycin was used to induce differentiation for three days, and the medium was changed every day.

### 2.7. Quantitative real-time PCR

All the cells were pipetted into 1 mL of TRIpure solution (EP013, ELK Biotechnology), and 250 µL of chloroform was added to it, mixed well, and allowed to stand still for 5 min on ice. The extracted RNA from the above step was then added to the reverse transcription reaction mixture for reverse transcription.

The primers used for actin were 5`-CTGAGAGGGAAATCGTGCGT-3` and 5`-CCACAGGATTCCATACCCAAGA-3`.

The primers used for GLUT4 were 5`-GTCAATACGGTCTTCACGTTGG-3` and 5`-ACATAGCTCATGGCTGGAACC-3`.

The primers used for UCP1 were 5`-GTACCAAGCTGTGCGATGTCC-3` and 5`-CGTGGTCTCCCAGCATAGAAG-3`.

Real-time PCR was performed by StepOne™ Real-Time PCR instrument using the EntiLink™ First Strand cDNA Synthesis Kit (EQ003, ELK Biotechnology). The formula used for the calculation was as follows: ΔCt = Ct value of the target gene-Ct value of the reference gene; ΔΔCt = ΔCt of the control group-ΔCt of the experimental group. The relative expression value of the target gene in the sample of the experimental group was calculated as 2^ΔΔCt^.

### 2.8. Data analysis

All data were analysed by SPSS 19.0 (SPSS/IBM 19.0, SPSS Inc., USA), and the results were expressed as mean ±SD. The difference between the two groups was determined using the student’s t-test. The difference was considered significant if p < 0.05.

## 3. Results

### 3.1. Olanzapine induced insulin resistance in rats, while the intervention of Gegen Qinlian Decoction improved it

**Figure 2.**
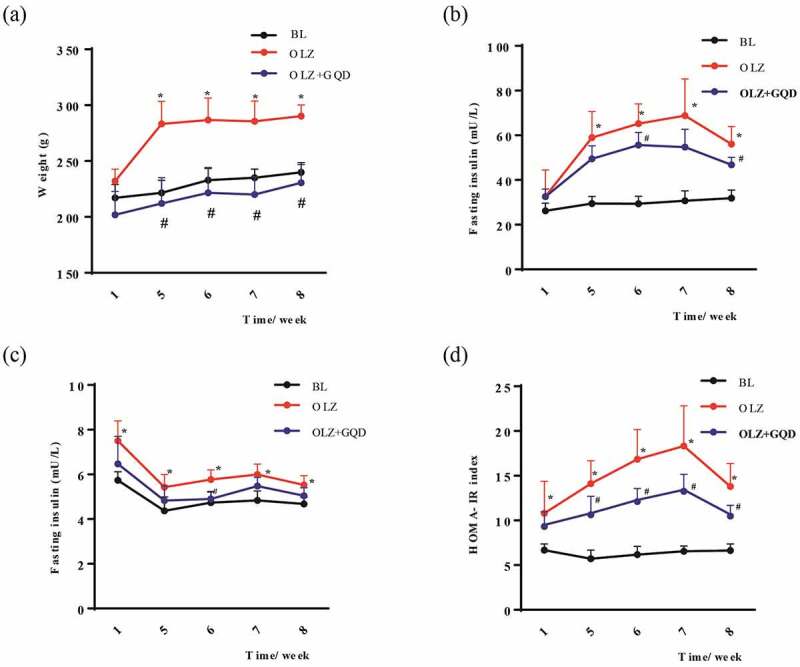
The effect of olanzapine and Gegen Qinlian Decoction on the body weight, blood glucose, fasting insulin concentration and HOMA-IR values in rats during a course of 8 weeks (a) Body weight in the first, fifth, sixth, seventh, and eighth week. (b)Fasting insulin concentration in the first, fifth, sixth, seventh, and eighth week. (c) Blood glucose levels in the first, fifth, sixth, seventh, and eighth week. (d) HOMA-IR in the first, fifth, sixth, seventh, and eighth week. The values are expressed as mean±SD (n = 6). For statistical significance, *p<0.05 shows the comparison to the BL group; ^#^p<0.05 shows the comparison to the OLZ group.

Long-term use of olanzapine can cause obesity, insulin resistance, worsen metabolic control or even induce diabetic ketoacidosis, and Gegen Qinlian Decoction has been widely used in the treatment of IR [2,21]. **A**s shown in Figure 2, the body weight of rats in the OLZ group increased significantly after daily administration of olanzapine for eight consecutive weeks when compared to the BL group and OLZ+GQD group (Figure 2(a)). Correspondingly, the blood glucose levels in the rats of the OLZ group were significantly higher than that of the BL group and the OLZ+GQD group (Figure 2(c)). The five weeks data of fasting insulin concentration and the HOMA-IR values of the rats in the OLZ group were significantly higher than that of the BL group and the OLZ+GQD group (Figure 2(b,d)).

**Figure 3.**
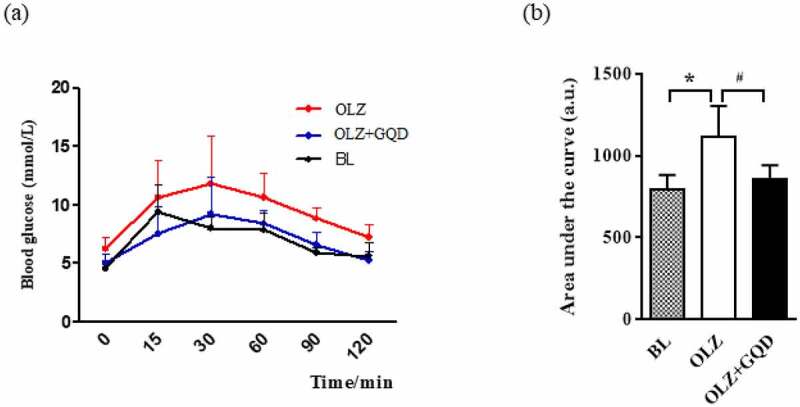
The effect of olanzapine and Gegen Qinlian Decoction on the OGTT. (a) OGTT was performed in the indicated groups after 8 weeks of gavage (b) the corresponding areas under the curve were calculated. The values are expressed as mean±SD (n = 6). For statistical significance, *p<0.05 shows the comparison to the BL group; ^#^p<0.05 shows the comparison to the OLZ group.

The OGTT was used to understand the function of pancreatic β-cells and also the body’s ability to regulate blood glucose levels. The results showed that the blood glucose concentration and AUC_mean_ in the OLZ group were significantly higher than that of the BL and OLZ+GQD groups. Also, the results of the OLZ+GQD and BL groups were found to be similar and are shown in Figure 3.

### 3.2. Pharmacokinetic study of olanzapine gavage

**Figure 4.**
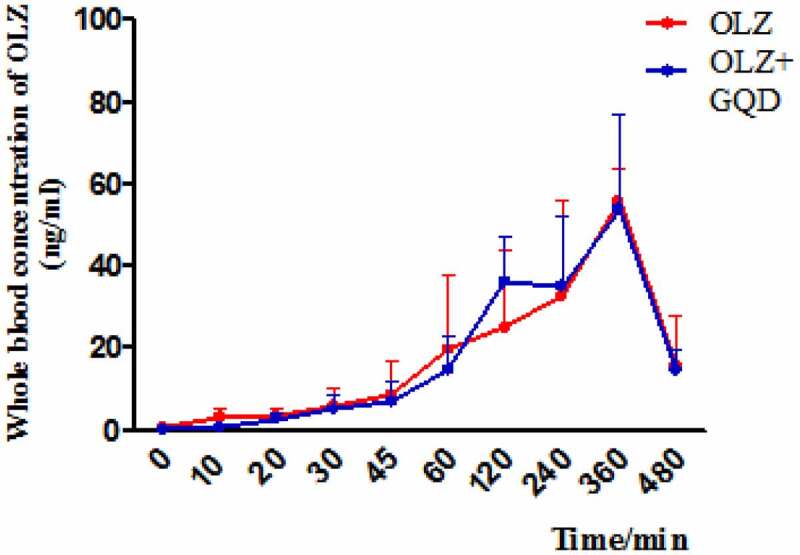
The time-concentration curves of different groups with olanzapine. The values are expressed as mean±SD (n = 6).

To explore whether GQD affects the metabolism of OLZ, we conducted pharmacokinetic study of olanzapine. As we can see, the calibration curve was linear with the assayed concentration range, where the mean regression value of the curve was *y* = 1.83*x*-0.513 (r^2 =^ 0.9916). The olanzapine concentration was calculated in rats at 0, 10, 20, 30, 45, 60, 120, 240, 360, and 480 min after its administration. The results are shown in Figure 4. No significant difference was found in the values of AUC (0-t), AUC (0-∞), t_1/2_, T_max_, C_max_ between the OLZ group and OLZ+GQD group, and the results are as shown in Table 1. The results proved that GQD did not affect the metabolism of OLZ in the body but improved olanzapine-induced IR.

**Table 1.** Pharmacokinetic parameters of olanzapine after a single oral dose treatment in female SD rats after o week gavage. AUC(0-∞), area under the concentration-time curve from zero to infinity; AUC(0-t), area under the concentration–time curve from zero to the last measurable plasma concentration;t_1/2_, elimination half-life; t_max_, time to reach peak plasma concentration; C_max_, peak plasma concentration

Group	AUC(0-t) (μg/L·h)	AUC(0-∞) (μg/L·h)	t_1/2_ (h)	T_max_ (h)	C_max_(μg/L)
OLZ	247.94 ± 106.46	374.77 ± 200.05	5.00 ± 1.67	5.67 ± 0.82	56.80 ± 8.10
OLZ+GQD	260.25 ± 93.04	365.37 ± 135.81	4.51 ± 0.92	4.67 ± 2.07	56.47 ± 18.68

### 3.3. Thermogenic tissue activity in rats for glucose uptake as assessed by ^18^F-FDG-PET/CT

**Figure 5.**
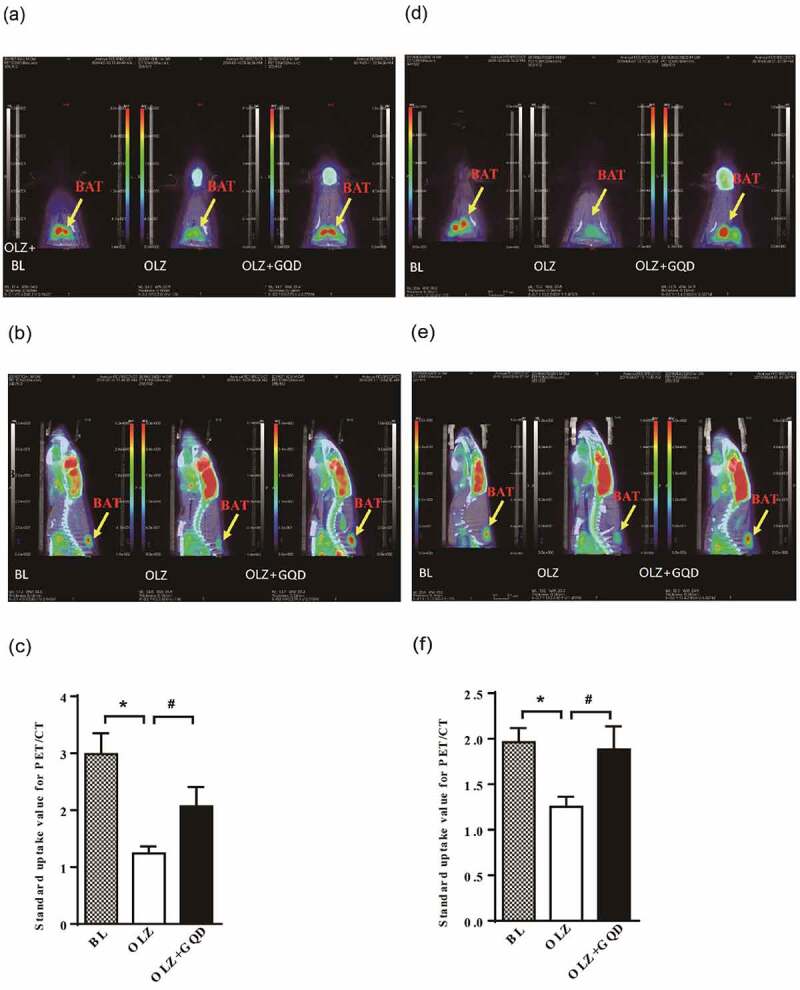
The effect of olanzapine and Gegen Qinlian Decoction on the activity of iBAT in rat models after 4 or 8 weeks gavage . (a) iBAT is marked in the picture at the positive position after 4th week of olanzapine administration. (b) The side position of iBAT after 4th week of olanzapine administration. (c) The positive position of iBAT after 8th week of olanzapine administration. (d) The side position of iBAT at the 8th week of olanzapine administration. (e) The standard uptake value of iBAT for 4 weeks. (f) The standard uptake value of iBAT for 8 weeks. In each case, the representative image shows n = 1 repeats. The values are expressed as mean±SD (n = 3). For statistical significance, *p<0.05 shows the comparison to the BL group; ^#^p<0.05 shows the comparison to the OLZ group.

A combination of PET and computed tomography (CT) scan, with the glucose analog ^18^F-fluorodeoxyglucose (^18^F-FDG) as a tracer, was used for diagnosis of neoplasms and their metastases, which showed a high glucose uptake by the supraclavicular tissue. The increased glucose uptake in the scapula area was thought to represent the presence of brown adipose tissue by ^18^F-FDG-PET/CT [24]. As shown in Figure 5, the brown adipose tissue of rats is observed in the interscapular region with different shapes in different positions. The positive position scan showed that iBAT was butterfly-shaped, which was consistent with the previous literature [25]. In Figure 5(a,b), it is observed that after four weeks of administration, the iBAT activity in the OLZ group rats was significantly reduced compared to the BL and OLZ+GQD group. This comparison between the groups was made using the standard uptake values (SUV) (The different colours observed in the iBAT area in the picture represent different SUVs). Figure 5(c,d) showed that after eight weeks of administration, the iBAT activity in the rats of the OLZ group was still significantly lower than that of the BL group and OLZ+GQD group. The SUV_mean_ of each group is shown in Figure 5(e) and

### 3.4. Olanzapine reduced the expression of UCP1 in both iBAT and scWAT, and the intervention of Gegen Qinlian Decoction improved it

**Figure 6.**
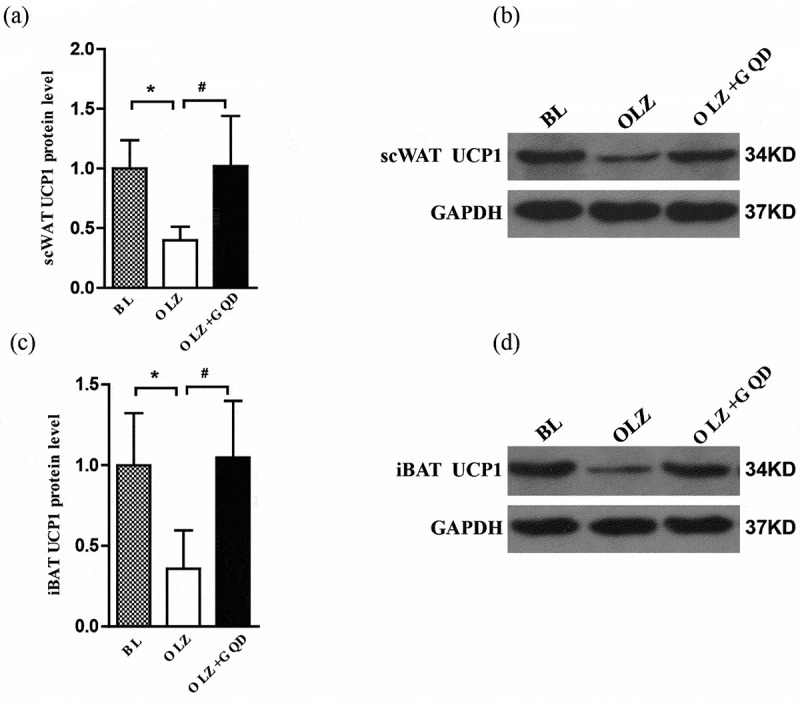
The effect of olanzapine and Gegen Qinlian Decoction on the expression of UCP1 in iBAT and scWAT in rat models after 8 weeks gavage. (a) UCP1 expression in scWAT. (b) Representative western blots of UCP1 expression in scWAT. (c) UCP1 expression in iBAT. (d) Representative western blots of UCP1 expression in iBAT. The corresponding control levels are arbitrarily assigned at a value of 1. The values are expressed as mean±SD (n = 6). For statistical significance, *p<0.05 shows a comparison to the BL group; ^#^p<0.05 shows a comparison to the OLZ group.

We evaluated the UCP1 protein content by Western blot analysis. As shown in Figure 6, the expression of UCP1 in both iBAT and scWAT in the OLZ group was significantly reduced compared to the BL and OLZ+GQD groups. This was also consistent with the results of the ^18^F-FDG-PET/CT.

### 3.5. Olanzapine reduced the expression of GLUT4 and PM GLUT4 in both iBAT and scWAT, and the intervention of Gegen Qinlian Decoction improved it

**Figure 7.**
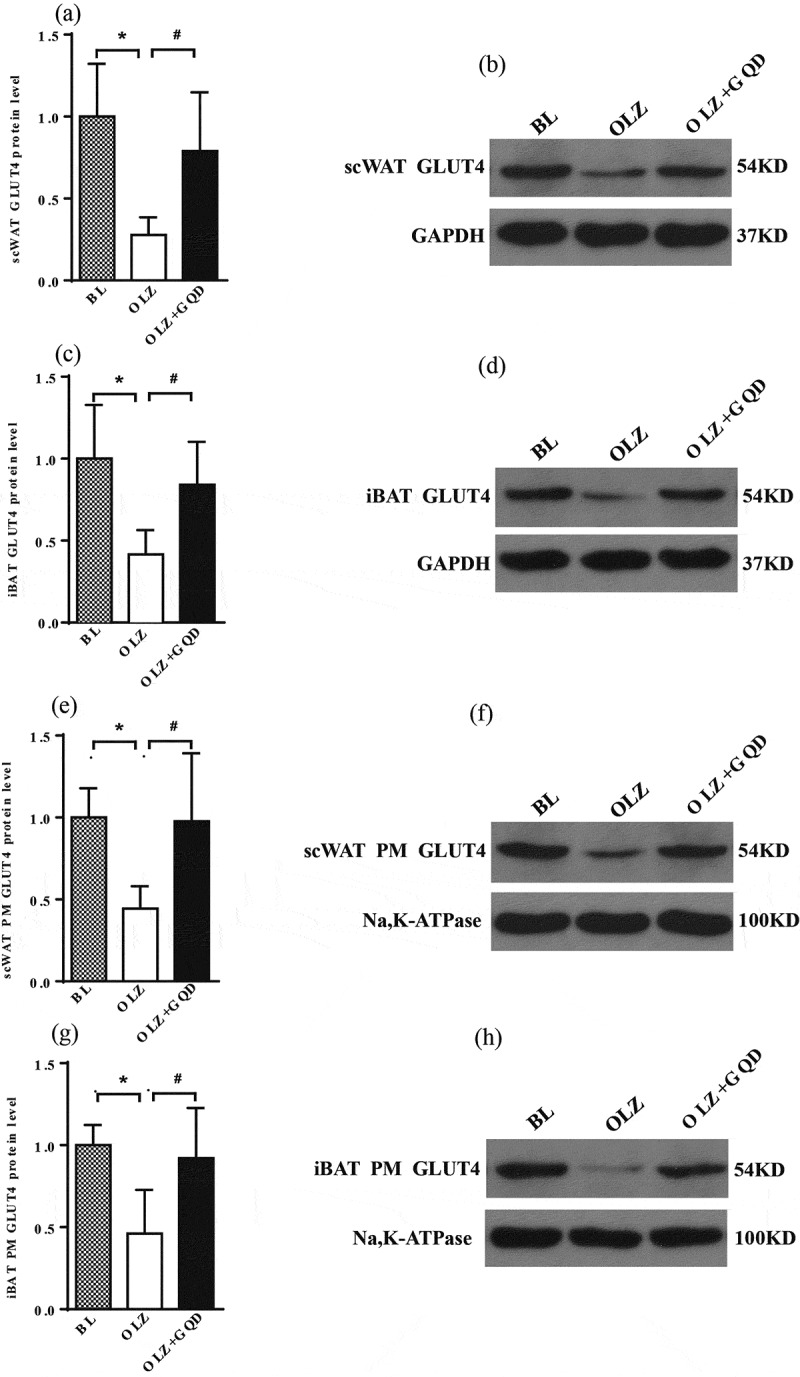
The effect of olanzapine and Gegen Qinlian Decoction on the overall expression and PM expression of GLUT4 protein in iBAT and scWAT in rat models after 8 weeks gavage (PM GLUT4: GLUT4 protein expression in plasma membrane and GLUT4: GLUT4 protein expression in whole-cell lysate). (a) GLUT4 expression in scWAT. (b) Representative western blots of GLUT4 expression in scWAT. (c) GLUT4 expression in iBAT. (d) Representative western blots of GLUT4 expression in iBAT. (e) The expression of GLUT4 in PM of the scWAT. (f) Representative western blots of PM GLUT4 expression in scWAT. (g) The expression of GLUT4 in PM of the iBAT. (h) Representative western blots of PM GLUT4 expression in iBAT. The corresponding control levels are arbitrarily assigned at a value of 1. The values are expressed as mea±SD (n = 6). For statistical significance, *p<0.05 shows the comparison to the BL group; ^#^p<0.05 shows the comparison to the OLZ group.

Glucose transporter 4 (GLUT4) carries glucose into the cells to maintain blood glucose balance. The insulin-stimulated glucose uptake by the GLUT4 transporter plays a central role in the whole-body glucose homoeostasis [26]. But when insulin binds to the receptor, GLUT4 translocates itself to the outer cell membrane, where it performs the best. Therefore, we tested the expression of GLUT4 protein in the plasma membrane (PM) of the iBAT and scWAT. The results showed that the expression of GLUT4 protein showed a significant decrease in the PM of the rats administered with olanzapine for eight weeks compared with BL group, while the GQD intervention could improve this decrease in GLUT4 expression caused by olanzapine, as shown in Figure 7.

### 3.6. Olanzapine reduced the expression of PM GLUT4 in both brown adipocytes and 3T3-L1 adipocytes, while the intervention of Gegen Qinlian Decoction improved it

**Figure 8.**
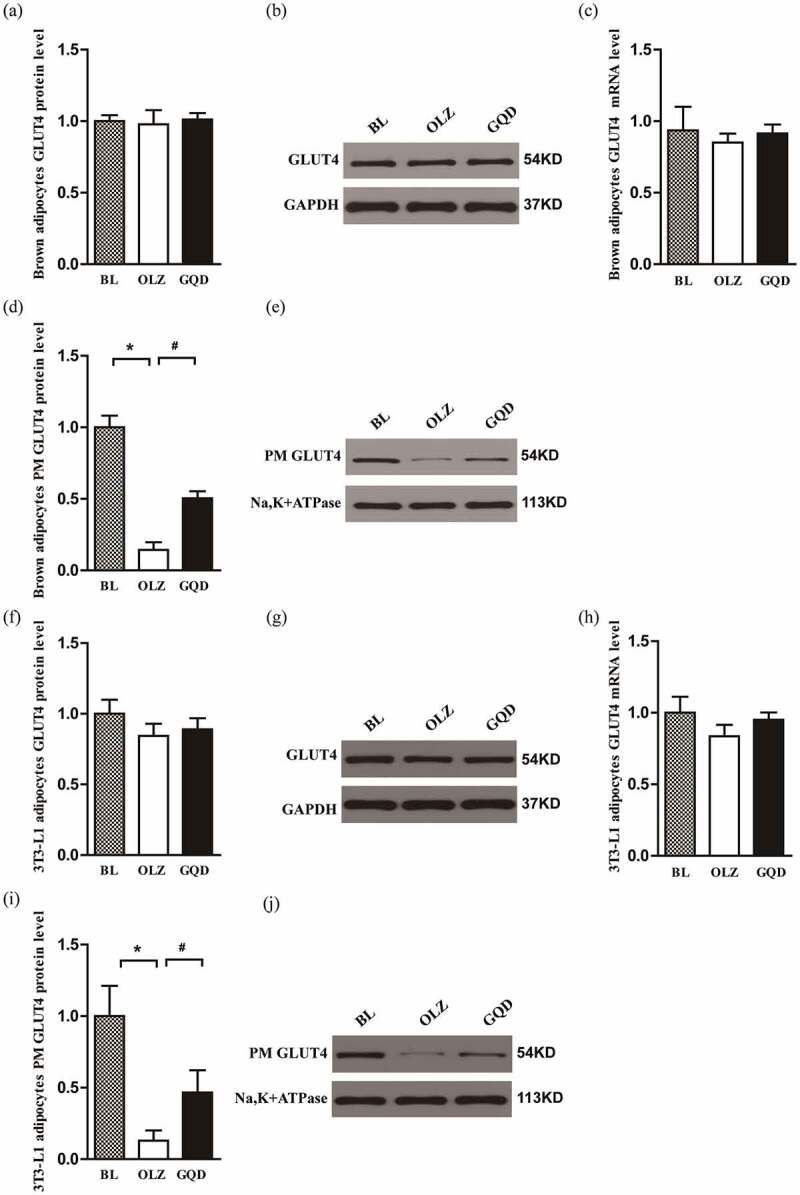
The effect of olanzapine and Gegen Qinlian Decoction on the overall expression and PM expression of GLUT4 protein in brown and 3T3-L1 adipocytes that were treated with 5 µM OLZ, GQD (puerarin, 100 mg•L^–1^; berberine, 100 mg•L^–1^; baicalin, 130 mg•L^–1^; glycyrrhizin, 20 mg•L^–1^) or OLZ+GQD for 48 h. (PM GLUT4: GLUT4 protein expression in plasma membrane and GLUT4: GLUT4 protein expression in whole-cell lysate). (a) GLUT4 expression in brown adipocytes. (b) Representative western blots of GLUT4 expression in brown adipocytes. (c) GLUT4 mRNA expression levels in brown adipocytes were determined by quantitative real-time PCR; (d) The expression of GLUT4 in PM of brown adipocytes. (e) Representative western blots of GLUT4 expression in PM of brown adipocyte. (f) GLUT4 expression in 3T3-L1 adipocytes. (g) Representative western blots of GLUT4 expression in 3T3-L1 adipocytes. (h) GLUT4 mRNA expression levels in 3T3-L1 adipocytes were determined by quantitative real-time PCR. (i) The expression of GLUT4 in PM of 3T3-L1 adipocytes. (j) Representative western blots of GLUT4 expression in PM of 3T3-L1 adipocytes. The corresponding control levels are arbitrarily assigned at a value of 1. The values are expressed as mean±SD (n = 3). For statistical significance, *p<0.05 shows the comparison to the BL group; ^#^p<0.05 shows the comparison to the OLZ group.

We also examined the effect of olanzapine on GLUT4 expression in brown and 3T3-L1 adipocytes and found that the total amount of GLUT4 was essentially unchanged between the groups. However, GLUT4 content in the plasma membranes varied between the groups. The PM GLUT4 was reduced significantly in the OLZ group compared to the BL group. Also, the Gegen Qinlian Decoction significantly increased the insulin-induced GLUT4 membrane translocation compared to that of the OLZ group, as shown in Figure 8.

### 3.7. Olanzapine reduced the expression of UCP1 in both brown adipocytes and 3T3-L1 adipocytes, while the intervention of Gegen Qinlian Decoction improved it

**Figure 9.**
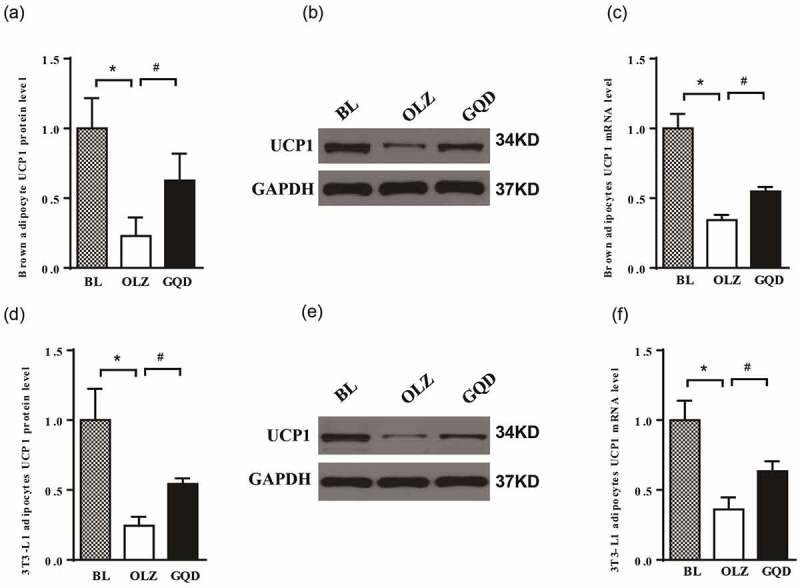
The effect of olanzapine and Gegen Qinlian Decoction on the expression of UCP1 in brown and 3T3-L1 adipocytes that were treated with 5 µM OLZ, GQD(puerarin, 100 mg•L^–1^; berberine, 100 mg•L^–1^; baicalin, 130 mg•L^–1^; glycyrrhizin, 20 mg•L^–1^) or OLZ+GQD for 48 h. (a) UCP1 expression in brown adipocytes. (b) Representative western blots of UCP1 expression in brown adipocytes. (c) mRNA expression levels of UCP1 in brown adipocytes were determined by quantitative real-time PCR. (d) UCP1 expression in 3T3-L1 adipocytes. (e) Representative western blots of UCP1 expression in 3T3-L1 adipocytes. (f) mRNA expression levels of UCP1 in 3T3-L1 adipocytes were determined by quantitative real-time PCR. The corresponding control levels are arbitrarily assigned at a value of 1. The values are expressed as mean±SD (n = 3). For statistical significance, *p<0.05 shows the comparison to the BL group; ^#^p<0.05 shows the comparison to the OLZ group.

**Figure 10.**
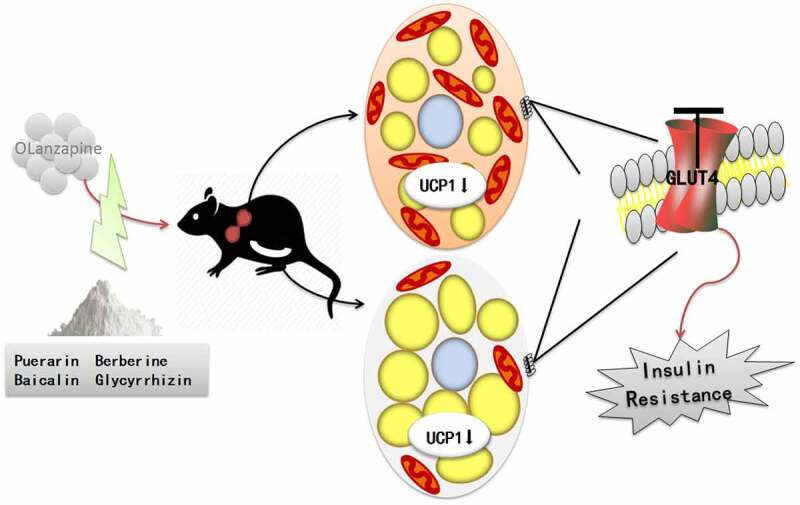
Olanzapine induces insulin resistance by depressing the expression of UCP1 and GLUT4 in iBAT and scWAT, Gegen Qinlian decoction attenuates these effects in rats.

To further investigate the mechanism of olanzapine and Gegen Qinlian Decoction on UCP1, we examined its expression in both brown and 3T3-L1 adipocytes by qRT-PCR and Western blotting analysis. As shown in Figure 9, the expression of UCP1 in the OLZ group decreased significantly compared to the BL group. Interestingly, the expression of UCP1 in the OLZ+GQD group was found to be elevated significantly compared to the OLZ group, which indicated that the Gegen Qinlian Decoction partially alleviated the inhibition of UCP1 caused by olanzapine. These findings demonstrated olanzapine induces IR by suppressing the expression of UCP1 and the GLUT4 translocation to the membrane in both iBAT and scWAT, GQD could attenuate this effect, as shown in Figure 10.

## 4. Discussion

Olanzapine has widely been prescribed as a maintenance treatment for severe schizophrenic illness but often produces severe metabolic side effects [27,28]. The data of weight, blood glucose, insulin resistance index, and OGTT in this article indicated that a long-term administration of olanzapine could cause metabolic syndromes, such as insulin resistance and impaired glucose tolerance. The PET-CT results indicated that the insulin resistance caused by olanzapine may be related to the inhibition of thermogenesis in BAT and impairment the glucose uptake. We also found that Gegen Qinlian Decoction could alleviate this inhibitory effect.

The thermogenic function of BAT is largely due to the expression of UCP1, which is a selectively expressed mitochondrial protein in BAT [29]. Apart from being a representative marker of beige/brite or brown adipocytes, thermogenic UCP-1 in white adipose tissues represents an emerging molecule in the regulation of energy metabolism and adipose inflammation [30]. Zhang et al. detected UCP1 in white fat in the groin and epididymis [31]. Previous studies also have shown that white adipose tissue can be induced by certain stimuli such as cold [32], exercise [11], to increase the expression of UCP1 and then show a ‘browning’ trend, thereby improving systemic metabolism. We tested the UCP1 content in the BAT and WAT of the IR rats, and the results showed that the UCP1 content was significantly decreased, which was also consistent with the previous studies [33,34].

AMP activated protein kinase(AMPK) pathway is a canonical route regulating energy homoeostasis. Olanzapine is known to change AMPK activity mostly as antagonists at H1 receptors [35]. Pgc-1α is a master transcriptional regulator of mitochondrial remodelling and driving UCP1 expression [36], and is tightly modulated by AMPK [37,38]. Zhang et al. found berberine increases expression of UCP1 in white and BAT and primary adipocytes via a mechanism involving AMPK and Pgc-1α [31]. UCP1 can lower the levels of reactive oxygen species (ROS) in isolated mitochondria, which can increase mitochondrial oxidative stress [39]. Study has shown that UCP1 deficiency causes the depletion of the brown fat respiratory chain, sensitizing mitochondria to calcium overload, thereby inducing dysfunction [40]. We can reasonably assume that olanzapine decreased expression of UCP1, then reduced the inhibitory effect existing on ROS production and increased mitochondrial oxidative stress, and membrane damage, ultimately damaging the thermogenic function of BAT.

Glucose uptake by the adipose tissue is principally regulated by GLUT4 [41]. GLUT4 protein was markedly reduced in adipose cells from mice with obesity-induced insulin resistance [42]. Poletto et al. showed soybean and sunflower oil-induced insulin resistance correlates with impaired GLUT4 protein expression and translocation in adipose tissue [43]. Our data exhibited GLUT4 expression, and GLUT4 in the PM of BAT was also significantly reduced in rats with olanzapine-induced IR, indicating that OLZ might affect membrane translocation of GLUT4, thereby impairing the glucose uptake of BAT. Previous study showed AMPK could influence the insulin-mediated increase in plasma membrane GLUT4 levels [44]. So we speculated olanzapine affects the translocation of GLUT4 may also through AMPK. AMPK is key signalling kinase that appears to regulate GLUT4 expression via the HDAC4/5-MEF2 axis and MEF2-GEF interactions resulting in nuclear export of HDAC4/5 in turn leading to histone hyperacetylation on the GLUT4 promoter and increased GLUT4 transcription [45]. Another finding showed integrin-linked kinase (ILK) blockade chronically inactivates AKT and shares with the exposed models some of the mechanisms that lead to reduced GLUT4 gene transcription [46]. Study showed olaznpine inhibited GLP1/GLP1R expressions in small intestine and pancreas, consequently lead to decreased insulin gene, including GLUT4, and ultimately dyslipidemia and glucose excursions [47]. Considering the complex mechanisms that affect GLUT4 expression and olanzapine may acted through a systemic action to influence glut4 expression in adipose tissue, we hypothesized olanzapine may be unable to influence intracellular GLUT4 expression in vitro, so the co-incubation of adipocyte with olanzapine didn’t change the intracellular GLUT4 expression significantly. Previous studies also indicate that olanzapine play a role mainly by affecting the expression of GLUT4 translocation to membrane. Wang et al. showed olanzapine decreased membrane GLUT4 protein expression and its ratio to total GLUT4 protein, whereas it minimally decreased total GLUT4 protein level [48]. Giovanni Tulipano et al. also showed, insulin and olanzapine increased the plasma membrane abundance of GLUT4, without changing GLUT4 content in the whole C2C12 cells [49].

In the present study, Gegen Qinlian Decoction could significantly reduce olanzapine-associated weight gain and fasting blood glucose elevations in the rat model, suggesting that GQD could improve these metabolic syndromes caused by olanzapine. Previous studies showed GQD ameliorates glucose metabolism disorders, and increases the sensitivity of the tissues to insulin in type 2 diabetic and HFD-induced IR rats [50]. GQD has been reported to improve HFD-induced insulin resistance, accompanied by increased expression of UCP1 and AMPKα in BAT [51]. A meta-analysis, including 499 patients, showed that GQD combined with metformin showed better efficacy in improving hyperglycaemia than metformin alone [18]. BAT activity in rats treated with OLZ combined with GQD was also increased compared with OLZ alone. To further explore the mechanism of GQD on BAT, we examined the expression levels of UCP1 *in vivo and vitro*, after the preventive effects of GQD were established during olanzapine treatment, and the results showed increased expression levels of UCP1. Given that olanzapine might decrease the expression of UCP1 through AMPK, it is also possible GQD might inhibit this effect of olanzapine through AMPK, and further lower the ROS production, improve the thermogenic function of BAT. On the other hand, the rats and the cells treated with OLZ and GQD showed increased expression of GLUT4 and membrane translocation of GLUT4 compared with OLZ alone, suggesting that GQD could partly alleviate olanzapine-induced IR by repairing the glucose uptake in adipose tissue. GQD reversed the GLUT4 alteration induced by olanzapine may also involving AMPK.

In summary, on the one hand, PET-CT results prompted olanzapine induces IR by suppressing BAT thermogenesis; on the other hand, olanzapine acts on the translocation process of GLUT4, inhibiting its ability to transport glucose. This results in impaired glucose uptake and inducing IR. But these data only partially explain the metabolic disturbance related to olanzapine and more in-depth mechanisms need to be explored. More importantly, we verified for the first time that GQD can alleviate IR caused by olanzapine effectively, as far as we knew, is a key aspect that affects patient compliance with medication. This may be helpful to patients taking olanzapine for a long time clinically.

## Data Availability

The data that support the findings of this study are available on request from the corresponding author, Weiyong Li. The data are not publicly available due to that could compromise the privacy of research participants.

## References

[1] Duggan L, Fenton M, Dardennes RM, et al. Olanzapine for schizophrenia. Cochrane Database Syst Rev. 2005 Apr 18;2. doi:10.1002/14651858.CD001359. PMC11781594 15846619

[2] Hegedus C, Kovacs D, Kiss R, et al. Effect of long-term olanzapine treatment on meal-induced insulin sensitization and on gastrointestinal peptides in female Sprague-Dawley rats. J Psychopharmacol. 2015 Dec;29(12):1271–1279. 26349558 10.1177/0269881115602952

[3] Carli M, Kolachalam S, Longoni B, et al. Atypical antipsychotics and metabolic syndrome: from molecular mechanisms to clinical differences. Pharmaceuticals. 2021 Mar;14(3):238. 33800403 10.3390/ph14030238 PMC8001502

[4] Aringhieri S, Carli M, Kolachalam S, et al. Molecular targets of atypical antipsychotics: from mechanism of action to clinical differences. Pharmacol Ther. 2018 Dec;192:20–41. 29953902 10.1016/j.pharmthera.2018.06.012

[5] Aringhieri S, Kolachalam S, Gerace C, et al. Clozapine as the most efficacious antipsychotic for activating ERK 1/2 kinases: role of 5-HT2A receptor agonism. Eur Neuropsychopharmacol. 2017 Apr;27(4):383–398. 28283227 10.1016/j.euroneuro.2017.02.005

[6] Men P, Yi Z, Li C, et al. Comparative efficacy and safety between amisulpride and olanzapine in schizophrenia treatment and a cost analysis in China: a systematic review, meta-analysis, and cost-minimization analysis. BMC Psychiatry. 2018 Sep;18(1). DOI:10.1186/s12888-018-1867-8. PMC6125952 30185173

[7] Poher AL, Altirriba J, Veyrat-Durebex C, et al. Brown adipose tissue activity as a target for the treatment of obesity/insulin resistance. Front Physiol. 2015 Jan;6. DOI:10.3389/fphys.2015.00004. PMC4311629 25688211

[8] Krause K. Novel aspects of white adipose tissue browning by thyroid hormones. Exp Clin Endocr Diab. 2020 Jun;128(6–7):446–449. 10.1055/a-1020-5354 31698480

[9] Hanssen MJW, Wierts R, Hoeks J, et al. Glucose uptake in human brown adipose tissue is impaired upon fasting-induced insulin resistance. Diabetologia. 2015 Mar;58(3):586–595. 25500952 10.1007/s00125-014-3465-8

[10] Chondronikola M, Volpi E, Borsheim E, et al. Brown adipose tissue improves whole-body glucose homeostasis and insulin sensitivity in humans. Diabetes. 2014 Dec;63(12):4089–4099. 25056438 10.2337/db14-0746 PMC4238005

[11] Stanford KI, Middelbeek RJW, Townsend KL, et al. Brown adipose tissue regulates glucose homeostasis and insulin sensitivity. J Clin Invest. 2013 Jan;123(1):215–223. 23221344 10.1172/JCI62308 PMC3533266

[12] Kuipers EN, Held NM, Panhuis WIH, et al. A single day of high-fat diet feeding induces lipid accumulation and insulin resistance in brown adipose tissue in mice. Am J Physiol-Endoc M. 2019 Nov;317(5):E820–E830. 10.1152/ajpendo.00123.2019 31386566

[13] Scheele C, Nielsen S. Metabolic regulation and the anti-obesity perspectives of human brown fat. Redox Biol. 2017 Aug;12:770–775. 28431377 10.1016/j.redox.2017.04.011 PMC5397125

[14] Shankar K, Kumar D, Gupta S, et al. Role of brown adipose tissue in modulating adipose tissue inflammation and insulin resistance in high-fat diet fed mice. Eur J Pharmacol. 2019 Jul;854:354–364. 30822393 10.1016/j.ejphar.2019.02.044

[15] Zhang Q, Lian J, He M, et al. Olanzapine reduced brown adipose tissue thermogenesis and locomotor activity in female rats. Prog Neuropsychopharmacol Biol Psychiatry. 2014 Jun;51:172–180. 24548587 10.1016/j.pnpbp.2014.02.003

[16] Kong H, Wang X-Q, Wang Q-G, et al. Effect of Puerarin on the pharmacokinetics of baicalin in gegen qinlian decoction in mice. Chin J Integr Med. 2018 Jul;24(7):525–530. 25908218 10.1007/s11655-015-1973-0

[17] Cao Z, Zeng Z, Wang B, et al. Identification of potential bioactive compounds and mechanisms of GegenQinlian decoction on improving insulin resistance in adipose, liver, and muscle tissue by integrating system pharmacology and bioinformatics analysis. J Ethnopharmacol. 2021 Jan;264:113289. 32846191 10.1016/j.jep.2020.113289

[18] Ryuk JA, Lixia M, Cao S, et al. Efficacy and safety of Gegen Qinlian decoction for normalizing hyperglycemia in diabetic patients: a systematic review and meta-analysis of randomized clinical trials. Complement Ther Med. 2017 Aug;33:6–13. 28735827 10.1016/j.ctim.2017.05.004

[19] Guo Y, Ding P-H, Liu L-J, et al. Gegen Qinlian Decoction attenuates high-fat diet-induced steatohepatitis in rats via gut microbiota. Evid Based Complement Alternat Med. 2018;2018:1–8. 10.1155/2018/7370891 PMC6323455 30671129

[20] Sui M, Chen G, Mao X, et al. Gegen Qinlian Decoction ameliorates hepatic insulin resistance by silent information regulator1 (SIRT1)-dependent deacetylation of Forkhead Box O1 (FOXO1). Med Sci Monit. 2019 Nov;25:8544–8553. 31719515 10.12659/MSM.919498 PMC6873633

[21] Zhang CH, Xu GL, Liu YH, et al. Anti-diabetic activities of Gegen Qinlian Decoction in high-fat diet combined with streptozotocin-induced diabetic rats and in 3T3-L1 adipocytes. Phytomedicine. 2013 Feb 15;20(3–4):221–229. 23219338 10.1016/j.phymed.2012.11.002

[22] Tian N, Wang J, Wang P, et al. NMR-based metabonomic study of Chinese medicine Gegen Qinlian Decoction as an effective treatment for type 2 diabetes in rats. Metabolomics. 2013 Dec;9(6):1228–1242.

[23] Tang Q, Li X, Song P, et al. Optimal cut-off values for the homeostasis model assessment of insulin resistance (HOMA-IR) and pre-diabetes screening: developments in research and prospects for the future. Drug Discov Ther. 2015 Dec;9(6):380–385. 26781921 10.5582/ddt.2015.01207

[24] Virtanen KA, Lidell ME, Orava J, et al. Functional brown adipose tissue in healthy adults (vol 360, pg 1518, 2009). N Engl J Med. 2009 Sep;361(11):1123. 10.1056/NEJMoa0808949 19357407

[25] Saely CH, Geiger K, Drexel H. Brown versus white adipose tissue: a mini-review. Gerontology. 2012;58(1):15–23. 21135534 10.1159/000321319

[26] Gustafson B, Hedjazifar S, Gogg S, et al. Insulin resistance and impaired adipogenesis. Trends Endocrin Met. 2015 Apr;26(4):193–200. 10.1016/j.tem.2015.01.006 25703677

[27] Townsend LK, Peppler WT, Bush ND, et al. Obesity exacerbates the acute metabolic side effects of olanzapine. Psychoneuroendocrino. 2018 Feb;88:121–128. 10.1016/j.psyneuen.2017.12.004 29241148

[28] Guo CL, Liu JX, Li HQ. Metformin ameliorates olanzapine-induced insulin resistance via suppressing macrophage infiltration and inflammatory responses in rats[J]. Biomed Pharmacother. 2021;133. DOI:10.1016/j.psyneuen.2017.12.004. 33217690

[29] Chouchani ET, Kazak L, Spiegelman BM. Spiegelman BM new advances in adaptive thermogenesis: UCP1 and beyond. Cell Metab. 2019 Jan;29(1):27–37. 30503034 10.1016/j.cmet.2018.11.002

[30] Li R-M, Chen S-Q, Zeng N-X, et al. Browning of abdominal aorta perivascular adipose tissue inhibits adipose tissue inflammation. Metab Syndr Relat Disord. 2017 Nov;15(9):450–457. 28934021 10.1089/met.2017.0074

[31] Zhang Z, Zhang H, Li B, et al. Berberine activates thermogenesis in white and brown adipose tissue. Nat Commun. 2014 Nov;5(1). DOI:10.1038/ncomms6493. 25423280

[32] Knudsen JG, Murholm M, Carey AL, et al. Role of IL-6 in exercise training- and cold-induced UCP1 expression in subcutaneous white adipose tissue. PLoS One. 2014 Jan;9(1):e84910. 24416310 10.1371/journal.pone.0084910 PMC3885654

[33] Song A, Dai W, Jang MJ, et al. Low- and high-thermogenic brown adipocyte subpopulations coexist in murine adipose tissue. J Clin Invest. 2020 Jan;130(1):247–257. 31573981 10.1172/JCI129167 PMC6934193

[34] Liu XM, Feng XY, Deng C, et al. Brown adipose tissue activity is modulated in olanzapine-treated young rats by simvastatin[J]. BMC Pharmacol Toxicol. 2020;21(1). DOI:10.1186/s40360-020-00427-0. PMC7325271 32605639

[35] Kim SF, Huang AS, Snowman AM, et al. Antipsychotic drug-induced weight gain mediated by histamine H-1 receptor-linked activation of hypothalamic AMP-kinase. Proc Natl Acad Sci U S A. 2007 Feb;104(9):3456–3459 17360666 10.1073/pnas.0611417104 PMC1805549

[36] Wu ZD, Puigserver P, Andersson U, et al. Mechanisms controlling mitochondrial biogenesis and respiration through the thermogenic coactivator PGC-1. Cell. 1999 Jul;98(1):115–124. 10412986 10.1016/S0092-8674(00)80611-X

[37] Besseiche A, Riveline JP, Gautier JF, et al. Metabolic roles of PGC-1 alpha and its implications for type 2 diabetes. Diabetes Metab. 2015 Nov;41(5):347–357. 25753246 10.1016/j.diabet.2015.02.002

[38] Lin JD, Handschin C, Spiegelman BM. Spiegelman BM metabolic control through the PGC-1 family of transcription coactivators. Cell Metab. 2005 Jun;1(6):361–370. 16054085 10.1016/j.cmet.2005.05.004

[39] Clarke KJ, Porter RK. Uncoupling protein 1 dependent reactive oxygen species production by thymus mitochondria. Int J Biochem Cell Biol. 2013 Jan;45(1):81–89. 23036787 10.1016/j.biocel.2012.09.023

[40] Kazak L, Chouchani ET, Stavrovskaya IG, et al. UCP1 deficiency causes brown fat respiratory chain depletion and sensitizes mitochondria to calcium overload-induced dysfunction. Proc Natl Acad Sci U S A. 2017 Jul;114(30):7981–7986. 28630339 10.1073/pnas.1705406114 PMC5544316

[41] Leto D, Saltiel AR. Regulation of glucose transport by insulin: traffic control of GLUT4. Nat Rev Mol Cell Bio. 2012 Jun;13(6):383–396. 22617471 10.1038/nrm3351

[42] Zhong XZ, Ke CF, Cai ZX, et al. LNK deficiency decreases obesity-induced insulin resistance by regulating GLUT4 through the PI3K-Akt-AS160 pathway in adipose tissue. Aging-Us. 2020 Sep;12(17):17150–17166. 10.18632/aging.103658 PMC7521507 32911464

[43] Poletto AC, Anhe GF, Eichler P, et al. Soybean and sunflower oil-induced insulin resistance correlates with impaired GLUT4 protein expression and translocation specifically in white adipose tissue. Cell Biochem Funct. 2010 Mar;28(2):114–121. 20087847 10.1002/cbf.1628

[44] Richter EA, Hargreaves M. EXERCISE, GLUT4, AND SKELETAL MUSCLE GLUCOSE UPTAKE. Physiol Rev. 2013 Jul;93(3):993–1017. 23899560 10.1152/physrev.00038.2012

[45] Vlavcheski F, Den Hartogh DJ, Giacca A, et al. Amelioration of high-insulin-induced skeletal muscle cell insulin resistance by resveratrol is linked to activation of AMPK and restoration of GLUT4 translocation. Nutrients. 2020 Apr;12(4):914. 10.3390/nu12040914. PMC7230755 32230718

[46] Hatem-Vaquero M, Griera M, Garcia-Jerez A, et al. Peripheral insulin resistance in ILK-depleted mice by reduction of GLUT4 expression[J]. J Endocrinol. 2017;234(2):115–128. 28490443 10.1530/JOE-16-0662

[47] Li DJ, Yue Q, Liu L, et al. Brexpiprazole caused glycolipid metabolic disorder by inhibiting GLP1/GLP1R signaling in rats[J]. Acta Pharmacol Sin. 2021;42(8):1267–1279. 33976388 10.1038/s41401-021-00680-x PMC8285380

[48] Wang CX, Wang CL, Ren LY, et al. The protein kinase D1-mediated inflammatory pathway is involved in olanzapine-induced impairment of skeletal muscle insulin signaling in rats[J]. Life Sci. 2021;270:119037. 33497738 10.1016/j.lfs.2021.119037

[49] Tulipano G, Spano P, Cocchi D. Effects of olanzapine on glucose transport, proliferation and survival in C2C12 myoblasts[J]. Mol Cell Endocrinol. 2008;292(1–2):42–49. 18514390 10.1016/j.mce.2008.04.010

[50] Li YM, Fan XM, Wang YM, et al. Therapeutic effects of Gegen Qinlian decoction and its mechanism of action on type 2 diabetic rats. Acta Pharm Sin B. 2013;48(9):1415–1421. https://24358775

[51] Zhang XQ, Xu WH, Xiao X, et al. Molecular mechanism of Gegen Qinlian Decoction in promoting differentiation of brown adipose tissue to improve glucose and lipid metabolism disorders in diabetic rats. Chin J Chin Mater Med. 2021;46(17):4462–4470. 10.19540/j.cnki.cjcmm.20210524.403 34581051

